# Magnetic resonance imaging highlights the meningeal involvement in steroid responsive meningitis-arteritis and suggests the inflammation of the surrounding tissues (70 cases)

**DOI:** 10.3389/fvets.2022.957278

**Published:** 2022-08-19

**Authors:** Carlotta Remelli, Alba Martello, Alessia Valentini, Barbara Contiero, Marco Bernardini

**Affiliations:** ^1^Department of Animal Medicine, Productions and Health, University of Padua, Legnaro, Italy; ^2^Anicura I Portoni Rossi Veterinary Hospital, Zola Predosa, Bologna, Italy

**Keywords:** steroid-responsive meningitis-arteritis (SRMA), dog, magnetic resonance imaging (MRI), cervical spine, polyarthritis, myositis, cerebrospinal fluid (CSF), central nervous system (CNS)

## Abstract

**Introduction:**

Steroid-responsive meningitis-arteritis (SRMA) is an immune-mediated disorder of young dogs for which there is no definitive ante-mortem diagnostic test. Magnetic Resonance Imaging (MRI) can be used to explore other differentials and extensive reports about its usefulness in the diagnosis of SRMA are lacking. The aims of this study were to retrospectively investigate the characteristics of MRI studies of the cervical spine of dogs diagnosed with SRMA and to compare the diagnostic capability of MRI obtained with low-field and high-field units.

**Materials and methods:**

This is a double center, retrospective case series. Databases were searched between 2008 and 2021 for dogs with a diagnosis of SRMA. Dogs were included if the following criteria were fulfilled: a diagnosis of cervical SRMA, results of CSF analysis, and MRI of the cervical spine available for re-evaluation.

**Results:**

Seventy cases were selected. MRI abnormalities were found in 69 cases (98.6%). Enhancement of the meninges, nerve roots, synovium of the articular facets and paravertebral muscles was present in 61 (87.1%), 10 (14.3%), 34 (48.6%), and 34 (48.6%) cases, respectively, when considering all MRI. In the low-field MRI, enhancement of these structures was present in 45 (90%), 4 (8%), 21 (42%) and 23 (46%) cases, respectively. In the high-field MRI, enhancement of these structures was present in 16 (80%), 6 (30%), 13 (65%) and 11 (55%) cases, respectively. Fat suppressed T1W images showed meningeal enhancement better than T1W images. When all the MRIs were considered, a significant increase in cell count of the cerebrospinal fluid was found between the three groups based on the meningeal MRI score (*p* = 0.001). In cases with no meningeal enhancement but enhancement of synovium of the articular facets and/or muscles a significantly lower cerebrospinal fluid cell count was present (*p* = 0.043), when considering all MRIs.

**Conclusions:**

The most frequent detection on cervical MRI of dogs affected by SRMA is meningeal enhancement, often accompanied by enhancement of the synovium of the articular facets and/or muscular enhancement. Both low-field and high-field MRI have good diagnostic capability but the latter enables a more thorough investigation thanks to specific sequences. MRI is useful as a complementary tool to cerebrospinal fluid analysis.

## Introduction

Steroid-responsive meningitis-arteritis (SRMA) is an immune-mediated disorder commonly recognized in dogs (1). Even though SRMA typically affects dogs of 6–18 months of age (1, 2) SRMA is also described in older dogs (3–6). Any breed can be affected, with a prominent predisposition in Beagles, Bernese Mountain Dogs, Border Collies, Boxers, English Springer Spaniels, Jack Russell Terriers, Nova Scotia Duck Tolling Retrievers, Weimaraners and Whippets (1, 7–9). An acute form and a chronic form of SRMA may occur. The former is characterized by cervical pain and stiffness, often associated with stiff gait and fever, the latter by ataxia, paresis, and spinal nerve deficits as a consequence of disease progression to the spinal cord (1, 10). SRMA-affected dogs may also show myositis and polyarthritis (11–13).

There are no definitive ante-mortem diagnostic tests for SRMA (14). Nowadays the diagnosis is based on a patient's particular signalment, medical history, neurological examination, bloodwork and, above all, cerebrospinal fluid (CSF) analysis. In the acute form CSF commonly presents a neutrophilic pleocytosis often characterized by more than 500 cells/μl in addition to an elevated protein concentration, whereas in the chronic form CSF is usually characterized by a mixed, less severe pleocytosis with a normal or mildly elevated total protein concentration (1, 15). Magnetic resonance imaging (MRI) of the cervical spine of dogs suspected to be affected by SRMA may show features suggesting meningeal inflammation, such as meningeal enhancement in T1-weighted (T1W) images after intravenous injection of a gadolinium-based contrast agent (GBCAs) (1, 16). Furthermore, MRI may reveal muscular abnormalities, such as T2-weighted (T2W) and short tau inversion recovery (STIR) hyperintensity (13, 16), as well as contrast enhancement (16). T2W hyperintensity or contrast enhancement inside the spinal cord may signal lesion progression (16). MRI is mainly considered useful to explore other differentials (16). Nevertheless, extensive reports about the usefulness of MRI in the diagnosis of SRMA are lacking.

The aims of this retrospective study were (1) to investigate the characteristics of MRI studies of the cervical spine of dogs diagnosed to be affected by SRMA and (2) to compare the diagnostic capability of MRI obtained with low-field (LF) and high-field (HF) MRI units.

## Materials and methods

### Case recruitment criteria

The medical records of dogs from 2 veterinary institutions (the Veterinary Teaching Hospital of the University of Padua and the AniCura Portoni Rossi Veterinary Hospital) were searched from August 2008 to November 2021 for dogs with a diagnosis of SRMA. Dogs were included if the following criteria were fulfilled: a diagnosis of cervical SRMA, results of CSF analysis, and MRI of the cervical spine available for re-evaluation. Eventual cases with a diagnosis of SRMA in spite of a normal CSF were excluded and discussed separately. For dogs that had a second presentation during the study period the relative medical record and cervical MRI were similarly reviewed.

### Medical records review

Details regarding signalment (breed, sex and age) and history (duration of clinical signs and pharmacological treatments in a 10-day time span before referral) were recorded. The results of the neurological examinations were reviewed and the following parameters were considered: cervical pain and stiffness, stiff gait, paresis, ataxia, and cranial and spinal nerve deficits. When available, body temperature was reviewed considering a temperature higher than 39.5°C as pyrexia. Response to the treatment was also noticed, when the follow-up was available.

### CSF analysis review

Cases with no CSF differential count were excluded. Cell count (White Blood Cells (WBC)/μL), neutrophil percentage and albumin concentration were assessed. The reference intervals for CSF parameters are reported elsewhere (15). Samples were divided in four classes based on the increased cell count (>500, 101–500, 31–100, 6–30 WBC/μL); four classes based on neutrophil percentage (>60, 31–60, 10–30, <10%) and four classes based on albumin concentration (>300, 101–300, 30–100, <30 mg/dL).

### MR images review

MRI studies were performed under general anesthesia with a LF (0.22 Tesla) MRI scanner (MrVet, Paramed Medical Systems, Genoa, Italy) from August 2008 to March 2018 and with a HF (1.5 Tesla) MRI scanner (Vantage Elan, Canon Medical Systems Europe B.V., Netherlands) from April 2018 to November 2021. To be included in the study and considered for re-evaluation, MRI had to include at least pre- and post-administration of a GBCA T1W images, either with or without fat suppression (T1W FAT-SAT), acquired in transverse and sagittal planes, as well as T2W or STIR images in the transverse plane.

All MRI studies were reviewed by two veterinarians alongside a board-certified neurologist using a DICOM viewer (2020 Horos Project™). The post-processing subtraction technique was used on T1W images to highlight contrast agent uptake. Comparison between T1W and T1W FAT-SAT images, as well as between T2W and STIR images, was then made to highlight differences in post-contrast enhancement and muscular hyperintensity, respectively.

To evaluate the extent of the meningeal contrast agent uptake, MR images in both transverse and sagittal planes were evaluated. A thick and long (over more contiguous images) enhancement of the meninges was considered suggestive of meningeal inflammation. Contrast agent uptake of nerve roots, synovium of the articular facets and paravertebral muscles was only analyzed in the transverse plane. For each structure a subjective score from 0 to 2 was assigned by each observer at the level of both the vertebral body (from C1 to C7) and the intervertebral space (from C1-C2 to C6-C7), based on the degree of contrast enhancement (Table 1). A similar score was assigned at the same levels, based on T2/STIR muscle hyperintensity, with scores 0, 1 and 2 meaning absent, mild and marked muscular hyperintensity, respectively. Spinal cord parenchyma contrast enhancement was recorded, if present. Each MRI study was then labeled for each structure as 0 if no contrast enhancement was present, as 1 if contrast enhancement was present at least in one cervical level. Finally, MRIs were globally classified based on the distribution of T1 contrast enhancement as absent or involving one or more of the following cervical structures: meninges, synovium of the articular facets, and muscles.

**Table 1:** Key to T1/T1 FAT-SAT contrast enhancement rating for each cervical structure analyzed in the study.

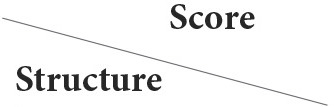	**0**	**1**	**2**
Meninges	No contrast enhancement	Mild and incomplete enhancement of the meningeal ring	Marked and complete enhancement of the meningeal ring
Nerve roots	No contrast enhancement or periradicular blood vessels enhancement	Mild nerve roots enhancement	Marked nerve roots enhancement
Synovium of the articular facets	No contrast enhancement	Mild synovial enhancement	Marked synovial enhancement
Muscles	No contrast enhancement	Mild muscular enhancement	Marked muscular enhancement

All the observers were aware of all data available in the medical reports. Images were independently analyzed by each observer. At each level, for a given structure to be labeled as “enhancing,” all the observers had to agree about the presence of the enhancement. Relatively to the entity of the enhancement, a discussion was carried on until a consensus between observers was reached.

A comparison of the above parameters between studies conducted with LF and HF MRI was then made to highlight possible differences due to the different magnetic strength.

### Crossmatch between CSF and MR images

The degree of meningeal enhancement seen in MRI, as well as the distribution of enhancement among cervical structures, were matched with CSF cell count to determine any potential correlation, as a whole and then separately for each type of MRI.

### Statistical analyses

Numerical variables are presented as median, interquartile range (IQR) and range. Categorical variables are summarized as counts and percentages. Statistical analysis was performed with the statistical software MedCal^®^ (Version 12.6.1.0, Software bvba). A *p*-value <0.05 was considered significant for all tests. A two proportion z-test for equality of two percentages was performed to (1) evaluate if a significant difference was present between LF and HF MRI in the detection of meningeal, nerve root, synovium of the articular facets, muscle, and spinal cord parenchyma enhancement; (2) evaluate if a significant difference was present between the incidence of synovium of the articular facets enhancement and muscular enhancement using LF and HF MRI and (3) estimate if the increased conspicuity of the meningeal contrast uptake shown through the subtraction technique with LF MRI was significantly different compared to that seen with HF MRI. A Kruskal-Wallis non-parametric test was performed to evaluate if significant differences were present between the CSF cell count in the three MRI groups based on the degree of meningeal enhancement (0 = absent, 1 = mild, 2 = marked), both on the total number of MRI and then on the LF and HF MRI individually. Cases were then grouped depending on the distribution of enhancement between cervical structures, and their CSF cell count distribution was studied with a Kruskal-Wallis non-parametric test, both on the total number of MRI and on LF and HF MRI separately. Three MRI patterns of enhancement were identified for this test: cases with meningeal and synovium of the articular facets and/or muscular enhancement (group 1), cases with meningeal enhancement alone (group 2), and cases with synovium of the articular facets and/or muscular enhancement but without meningeal enhancement (group 3).

A Fisher exact test was performed to analyze if meningeal enhancement on MRI was predictive of CSF abnormality (2 × 2 contingency table). In this test the cases with normal CSF were also included.

## Results

### Case recruitment criteria

Ninety-three cases were included after the first search based on a diagnosis of SRMA in the medical records. Of these, 4 cases were excluded because of the absence of a CSF differential cell count, and 17 cases because of an incomplete MRI protocol. Two cases had a normal CSF and were then excluded; they will however be separately discussed. Seventy cases were therefore included in the study. Two dogs fulfilling the inclusion criteria presented twice, therefore results for breed and sex represent a total of 68 dogs.

### Medical records review

The most represented breeds were Boxers (*n* = 14; 20.6%), mixed breeds (*n* = 14; 20.6%), and Bernese Mountain Dogs (*n* = 10; 14.7%). Table 2 lists all the breeds included in the study. There were 34 males (50%) and 34 females (50%). Duration of the clinical signs was <4 days in 25 dogs, 4–10 days in 24 dogs, and more than 10 days in 21 dogs. Median duration of the clinical signs before referral was 6.5 days (IQR 11.75; range 1–60 days). Four cases were confirmed relapses based on the CSF analysis and 6 cases were suspected relapses based on the history. Median age at the first presentation was 10 months (IQR 5.5; range 3–67 months). In the 10 day period before referral, 53 dogs (75.7%) had received one or more drugs including glucocorticoids (*n* = 9), non-steroidal anti-inflammatory drugs (NSAIDs) (*n* = 27), opioids (*n* = 24) and antimicrobials (*n* = 24). At neurological examination, 50 dogs (71.4%) had both cervical pain and stiffness, 12 dogs (17.1%) manifested only cervical pain and 4 dogs (5.7%) had only cervical stiffness; in 4 dogs (5.7%) cervical pain or stiffness were not detected on the neurological examination but were reported in the history. Twelve dogs (17.1%) showed paresis and 14 dogs (20%) showed ataxia. Other deficits detected on the neurological examination were: spinal nerve reflex abnormalities (*n* = 8), proprioceptive deficits (*n* = 6), cranial nerve deficits (*n* = 3), and an abnormal menace response (*n* = 3). Body temperature data was available for 32 dogs, 24 of which (75%) were pyretic. Response to the treatment was documented in 57 cases (81.4%) and was reported to be good in all of them.

**Table 2:** Breeds included in the study.

**Breeds**	**Number (%)**
Boxer	14 (20.6%)
Mixed breed dog	14 (20.6%)
Bernese mountain dog	10 (14.7%)
Weimaraner	6 (8.8%)
Beagle	4 (5.9%)
Golden retriever	3 (4.4%)
Dachshund, pitbull terrier	2 (2.9%)
Border collie, bull mastiff, dogo argentino, german shepherd dog, labrador retriever, maremma sheepdog, nova scotia duck tolling retriever, pembroke welsh corgi, pointer, poodle, schnauzer, spitz, whippet	1 (1.47%)

### CSF analysis review

Sixty-three (90%) CSF samples were collected from the cerebellomedullary cistern and 7 (10%) from the lumbar subarachnoid space. Median cell count was 945 WBC/μL (IQR 1623.75; range 7–6,133 WBC/μL). Forty-five (64.3%) CSF samples showed a very marked pleocytosis, with more than 500 WBC/μL and a median cell count of 1,504 WBC/μL (IQR 1515; range 509-6133 WBC/μL). In 65 (92.9%) samples, more than 60% of the cells were neutrophils, with a median neutrophil percentage of 81% (IQR 15, range 61–98%). The results of CSF analysis (grouped by cell count, neutrophil percentage and albumin concentration) are summarized in Table 3.

**Table 3:** CSF analysis results.

**Parameters evaluated**	**Number (%)**
**Cell count**	
>500 WBC/ μL	45 (64.3%)
101–500 WBC/μL	11 (15.7%)
31–100 WBC/μL	5 (7.1%)
6–30 WBC/μL	9 (12.9%)
**Neutrophil percentage**	
>60%	65 (92.9%)
31–60%	4 (5.7%)
10–30%	1 (1.4%)
<10%	0 (0.0%)
**Albumin concentration**	
>300 mg/dL	11 (15.7%)
101–300 mg/dL	9 (12.9%)
30–100 mg/dL	45 (64.3%)
<30 mg/dL	5 (7.1%)

### MR images review

**Figure 1:**
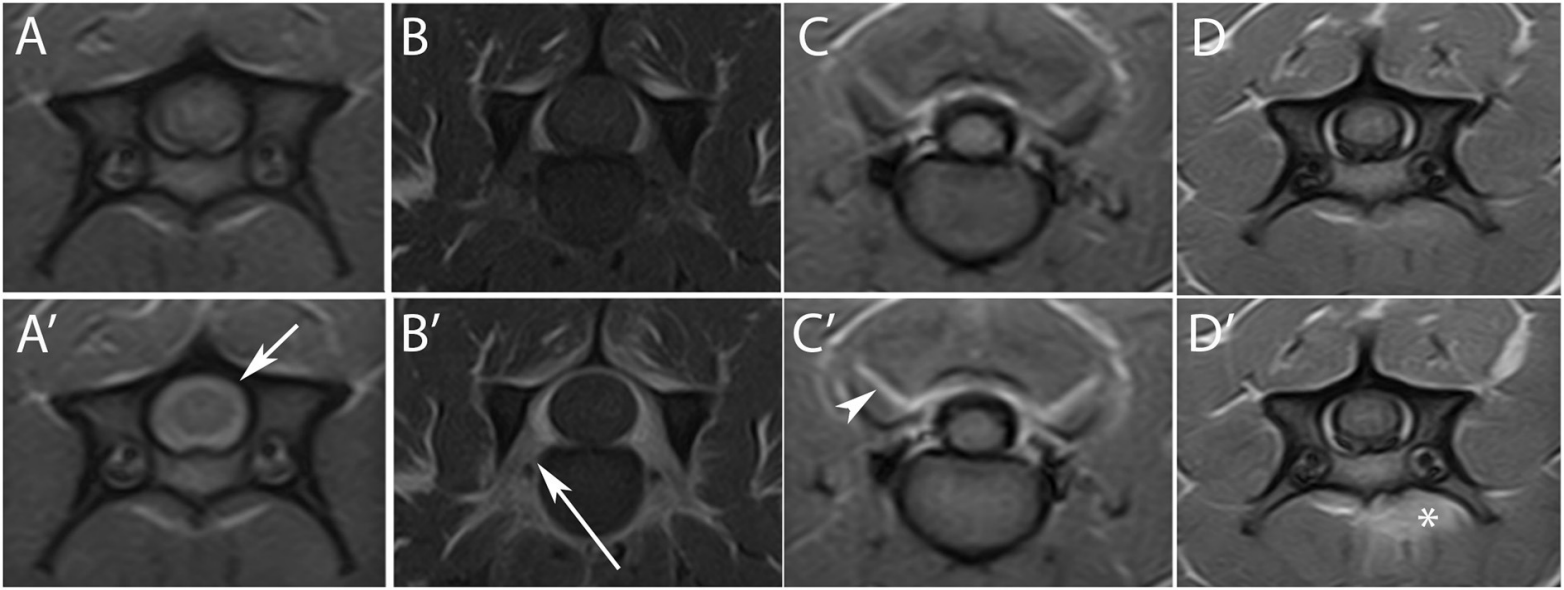
Transverse T1W images pre- (upper row) and post- (lower row) contrast agent administration, at level of C4 **(A,A',D,D')**, C5–C6 **(B–B')**, and C2–C3 **(C–C')**. A marked contrast enhancement is evident at the level of the meninges [**(A')**—short arrow], nerve roots [**(B')**—long arrow], synovium of the articular facets [**(C')**—arrowhead], and muscles [**(D')**—asterisk].

Sixty-nine (98.6%) MRI studies showed contrast enhancement of one or more structures. T1W post-contrast images demonstrated enhancement of meninges in 61 cases (87.1%), nerve roots in 10 (14.3%), synovium of the articular facets in 34 (48.6%) and paravertebral muscles in 34 (48.6%). Marked contrast enhancement (score 2) was present in 33 studies (45.8%) in at least one compartment, including meninges (*n* = 15), nerve roots (*n* = 3), synovium of the articular facets (*n* = 11) and paravertebral muscles (*n* = 10) (Figure 1). Moreover, spinal cord parenchymal contrast enhancement was present in 10 studies (14.3%) and was always associated with meningeal enhancement. Distribution of meningeal contrast enhancement in the cervical spine is summarized in Table 4. In the 8 MRI studies where both STIR and T2W sequences were available, muscular lesions were detected in both modalities in 5 cases, even easily detectable on STIR sequence, and were not seen in any in the remaining 3. The 34 cases demonstrating hyperintense muscular areas in either T2W or STIR sequences also showed T1W contrast enhancement of the same areas.

**Table 4:** Distribution of meningeal contrast enhancement in the cervical spine.

**Cervical level**	**Number (%)**
C1	32/63 (50.8%)
C1–C2	36/68 (52.9%)
C2	43/69 (62.3%)
C2–C3	49/70 (70.0%)
C3	53/70 (75.7%)
C3–C4	53/70 (75.7%)
C4	50/70 (71.4%)
C4–C5	48/69 (69.6%)
C5	42/69 (60.9%)
C5–C6	40/69 (58.0%)
C6	38/69 (55.1%)
C6–C7	37/67 (55.2%)
C7	33/63 (52.4%)

*For each cervical level, the percentage of cases with meningeal enhancement was calculated based on the number of images available at that level*.

**Figure 2:**
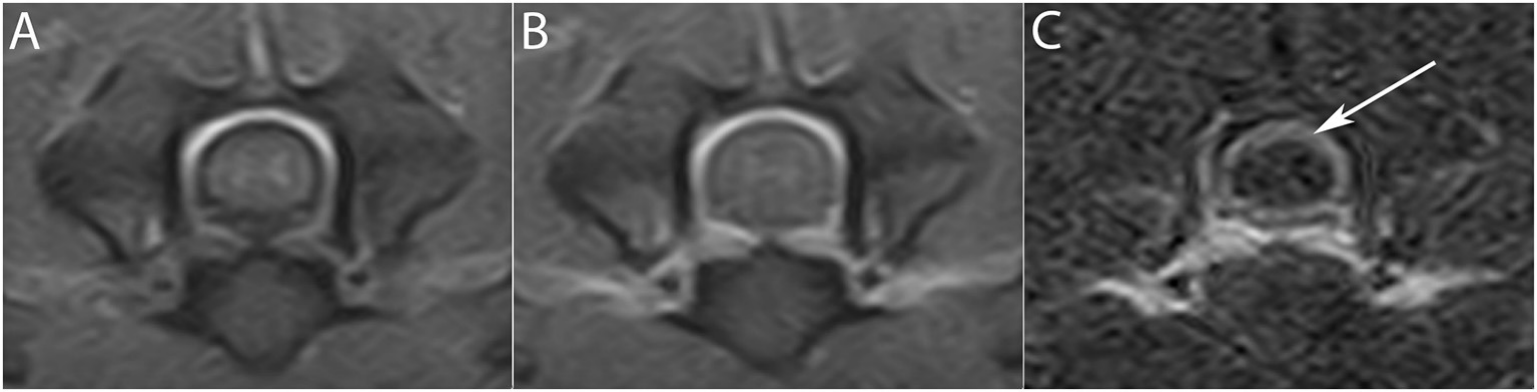
Subtraction technique in the detection of meningeal enhancement. **(A)** Pre- and **(B)** post-contrast transverse T1W image at the level of the C5–C6 intervertebral space. After subtraction **(C)**, a subtle meningeal enhancement not easily detectable in **(B)** is seen (arrow).

The post-processing subtraction technique increased the conspicuity of the enhancement at the meningeal level in 42 cases (60%) (Figure 2), in the synovium of the articular facets in 7 cases (10%) and in muscles in 1 case (1.4%). The incidence of contrast uptake was 48.6% for both the synovium of the articular facets and muscular structures. MRI patterns of alterations due to T1 contrast enhancement and their distribution between LF and HF MRI are summarized in Table 5.

**Table 5:** MRI patterns of alterations based on T1 contrast enhancement distribution between cervical structures.

**Enhancing structures**	**Total MRI**	**Low field MRI**	**High field MRI**
None (normal MRI)	1	1	0
Meninges	16	12	4
Synovium of the articular facets	1	1	0
Muscles	3	2	1
Meninges and synovium of the articular facets	18	13	5
Meninges and muscles	16	14	2
Synovium of the articular facets and muscles	4	1	3
Meninges, synovium of the articular facets and muscles	11	6	5
Total number of cases	70	50	20

T1W post-contrast images evaluated separately from the LF (50 cases) and HF (20 cases) MRI demonstrated enhancement of meninges in 45 (90%) and 16 (80%) cases, respectively, nerve roots in 4 (8%) and 6 (30%), respectively, synovium of the articular facets in 21 (42%) and 13 (65%), respectively, paravertebral muscles in 23 (46%) and 11 (55%), respectively, and in the spinal cord parenchyma in 9 (18%) and 1 (5%), respectively. No significant difference was found between LF and HF MRI in the identification of meningeal (*p* = 0.463), synovium of the articular facets (*p* = 0.140), paravertebral muscles (*p* = 0.677), and spinal cord parenchyma (*p* = 0.305) enhancement. A mild significant difference in the identification of nerve root enhancement (*p* = 0.046) was found.

The subtraction technique increased the conspicuity of contrast agent uptake at the level of the meninges in 34 (68%) LF and 8 (40%) HF MRI. In 5 (10%) LF MRI and in 3 (15%) HF MRI, the diagnosis would have been missed without the use of the subtraction technique. However, no significant difference in the utility of the subtraction technique at the level of the meninges was found between LF and HF MRI (*p* = 0.059).

Incidence of contrast uptake in the synovium of the articular facets was 42 and 65% and incidence of muscular contrast uptake was 46 and 55% in LF and HF MRI, respectively. However, no significant difference was found between LF and HF MRI in enhancement detection at the level of either synovium of the articular facets or muscles (*p* = 0.140 and *p* = 0.677).

**Figure 3:**
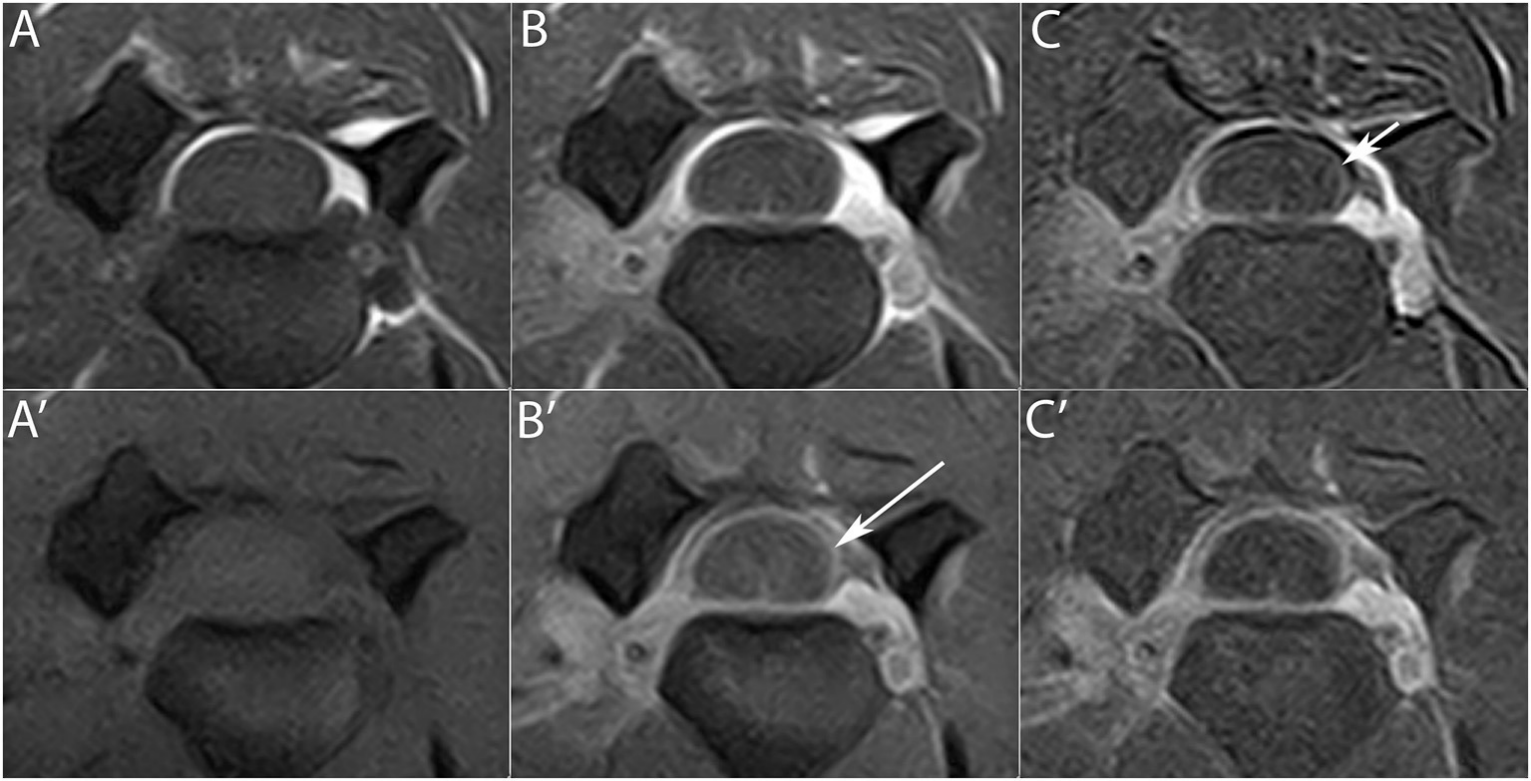
Transverse T1W (upper row) and T1 FAT SAT (lower row) images pre- **(A,A')** and post- **(B,B')** contrast administration at the level of the C3–C4 intervertebral space. Meningeal enhancement is visible on the T1W images only with the application of the subtraction technique **(C)** (short arrow), while it is easily detectable in T1W FAT SAT images **(B')** (long arrow). The subtraction technique in T1W FAT SAT images **(C')** is therefore not necessary.

Seven HF MRI had both T1W and T1W FAT SAT sequences available. In 4 of 5 MRI (80%), in which meningeal enhancement was present, the contrast uptake was seen better on the T1W FAT SAT images. Particularly, in 3 of these 4 cases meningeal enhancement was only easily detectable on T1W images through the application of the subtraction technique, whereas on T1W FAT SAT this was never necessary (Figure 3). Enhancement of the synovium of the articular facets was present in 5 MRI and in 2 (40%) of these the contrast uptake was seen better on the T1W FAT SAT images. In 1 (25%) of the 4 studies in which muscular enhancement was present, the contrast uptake was seen only on the T1W FAT SAT images.

### Crossmatch between CSF and MR images

**Figure 4:**
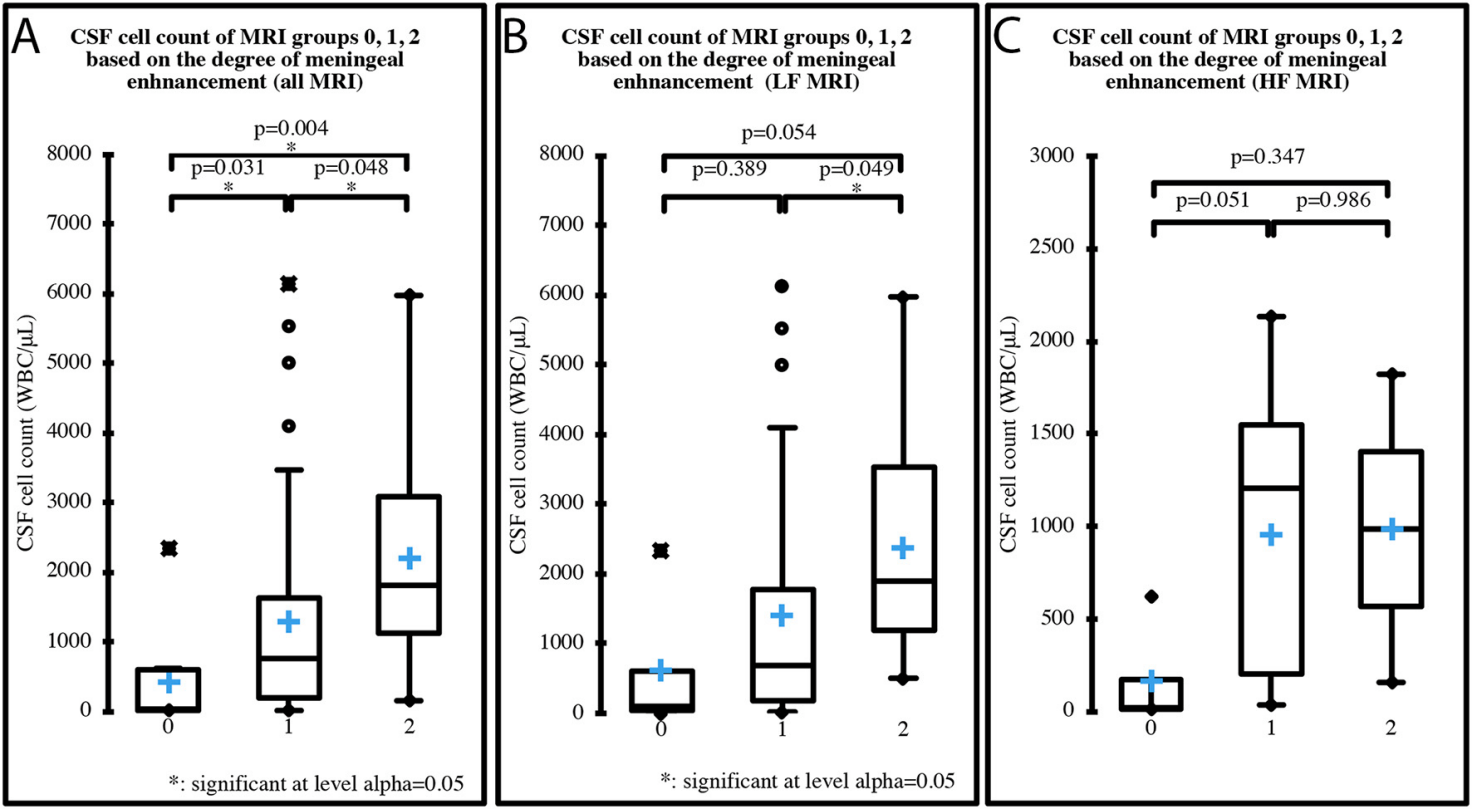
CSF cell count distribution in MRI groups based on the degree of meningeal enhancement (group 0 = absent, group 1 = mild, group 2 = marked), analyzing all 70 MRI **(A)**, the 50 LF MRI **(B)** and the 20 HF MRI **(C)**. Boxes contain values from 1st to 3rd quartile, lines inside boxes indicate median values, crosses inside boxes indicate mean values, endpoints of vertical lines are proportional to the interquartile-deviation and dots outside “whiskers” represents outlier values.

Median CSF cell count of the three groups based on the degree of MRI meningeal enhancement was 39 WBC/μL (IQR 581; range 7–2,341) in group 0, 757 WBC/μL (IQR 1435.8; range 16–6,133) in group 1, and 1,820 WBC/μL (IQR 1951.5; range 156–5,976) in group 2, when all the MRI were considered. When only the LF MRI were considered, median cell count was 111 WBC/μL (IQR 558; range 7–2,341) in group 0, 682 WBC/μL (IQR 1595.8; range 16–6,133) in group 1 and 1,896 WBC/μL (IQR 2,334; range 509–5,976) in group 2. When only the HF MRI were considered, median cell count was 19.5 WBC/μL (IQR 156.5; range 15–620) in group 0, 1206.5 WBC/μL (IQR 1,344; range 35–2,133) in group 1 and 988 WBC/μL (IQR 832; range 156–1,820) in group 2. A significant increase in CSF cell count was found between each of the three groups when all the MRI were considered (*p* = 0.001) (Figure 4A), and between groups 1 and 2 when only the LF MRI were considered (*p* = 0.016) (Figure 4B). No significant differences were found between the groups when only the HF MRI were considered (*p* = 0.06) (Figure 4C).

**Figure 5:**
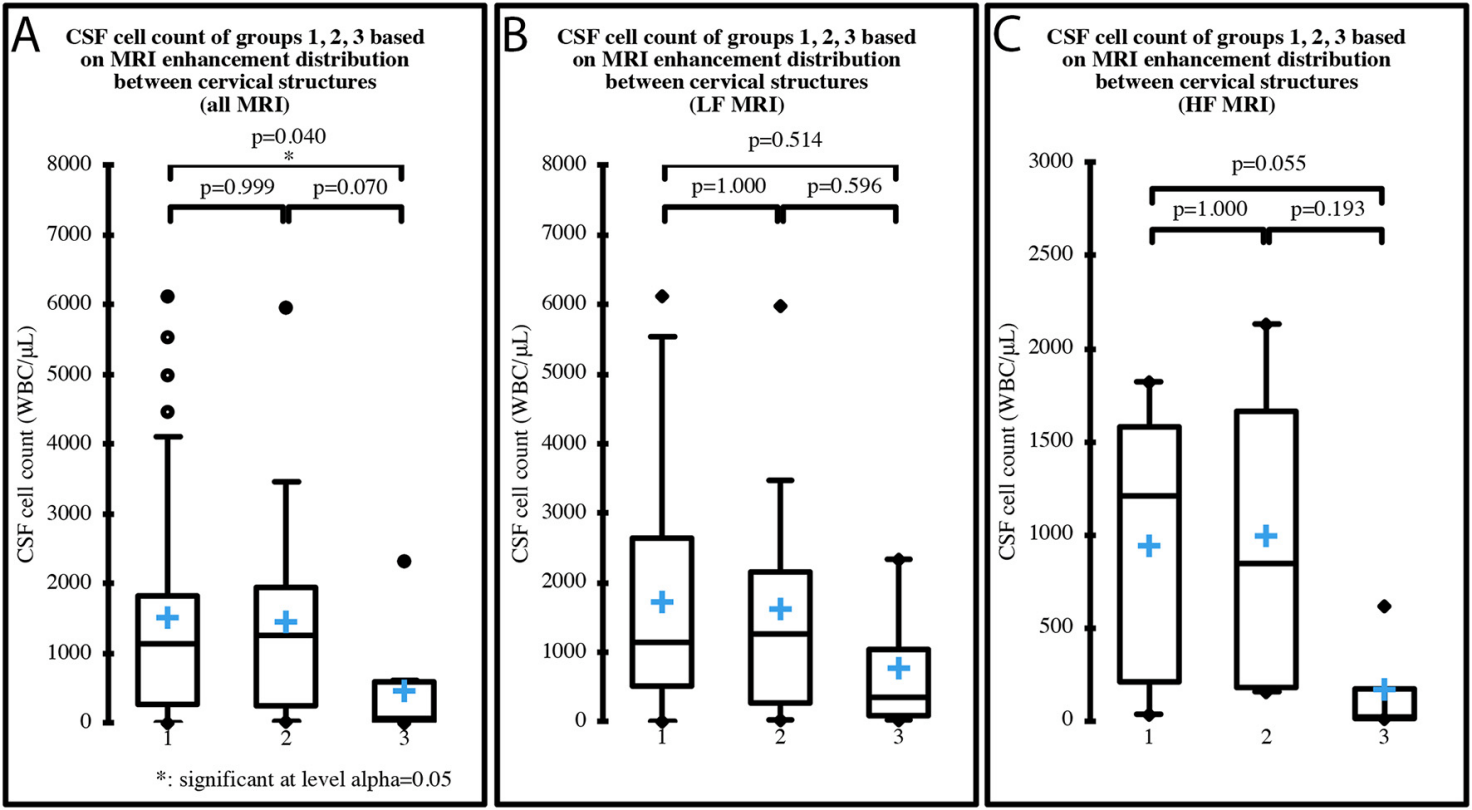
CSF cell count distribution in MRI groups based on the distribution of enhancement among cervical structures (group 1 = meningeal enhancement concurrently with synovium of the articular facets and/or muscular enhancement, group 2 = meningeal enhancement alone, group 3 = synovium of the articular facets and/or muscular enhancement). One case had normal MRI and was therefore not included in the analysis, which was performed on all 69 MRI **(A)**, on the 49 LF MRI **(B)** and on the 20 HF MRI **(C)**. Boxes contain values from 1st to 3rd quartile, lines inside boxes indicate median values, crosses inside boxes indicate mean values, endpoints of vertical lines are proportional to the interquartile-deviation and dots outside “whiskers” represents outlier values.

Median cell count of the three groups based on the MRI distribution of enhancement between cervical structures was 1,136 WBC/μL (IQR 1,554; range 16–6,133) in group 1, 1,262 WBC/μL (IQR 1,707; range 24–5,976) in group 2 and 75 WBC/μL (IQR 581.5; range 15–2,341) in group 3, when all the MRI were considered. When only the LF MRI were considered, median cell count was 1,136 WBC/μL (IQR 2,126; range 16–6,133) in group 1, 1,262 WBC/μL (IQR 1885.5; range 24–5,976) in group 2 and 354 WBC/μL (IQR 940; range 39–2,341) in group 3. When only the HF MRI were considered, median cell count was 1206.5 WBC/μL (IQR 1377.3; range 35–1,820) in group 1, 848 WBC/μL (IQR 1478.3; range 156–2,133) in group 2 and 19.5 WBC/μL (IQR 156.5; range 15–620) in group 3. One case had a normal MRI and was therefore not included in the analysis. A significant decrease in CSF cell count was found between group 1 and group 3, when all MRI were considered (*p* = 0.043) (Figure 5A). No significant differences were found between the groups when only LF (Figure 5B) or HF MRI (Figure 5C) were considered. MRI meningeal enhancement was not predictive of CSF abnormality (*p* = 0.260). MRI and CSF findings are summarized in Table 6.

**Table 6:** Crossmatch between MRI findings (columns) and CSF findings (rows).

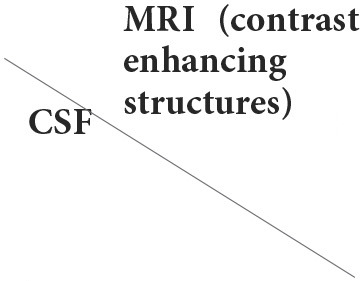	**None (normal MRI)**	**Meninges**	**Synovium of the articular facets**	**Muscles**	**Meninges and synovium of the articular facets**	**Meninges and muscles**	**Synovium of the articular facets and muscles**	**Meninges, synovium of the articular facets and muscles**
**Cell count**								
>500 WBC/μL	0	10	1	0	12	15	2	5
101–500 WBC/μL	0	4	0	1	3	0	0	3
31–100 WBC/μL	0	1	0	1	2	0	0	1
6–30 WBC/μL	1	1	0	1	1	1	2	2
**Neutrophil %**								
>60%	1	16	1	1	17	15	4	10
31–60%	0	0	0	1	1	1	0	1
10–30%	0	0	0	1	0	0	0	0
<10%	0	0	0	0	0	0	0	0
**Albumin concentration**								
>300 mg/dL	0	3	0	0	6	2	0	0
101–300 mg/dL	0	2	0	0	2	5	0	0
30–100 mg/dL	1	9	1	3	10	8	4	9
<30 mg/dL	0	2	0	0	0	1	0	2

*For each match, the number of cases is reported*.

## Discussion

Due to the retrospective nature of this paper, encompassing a 13-year period, a common gold standard diagnostic test for SRMA was not available. The diagnosis of SRMA was made relying not on a single parameter, but on a combination of patient's medical history of cervical pain, neurological examination, MRI and CSF results. Anyway, the white cell differential played an important role: 60% or more neutrophils on CSF cell count were found in all cases but 4. Two of this 4 cases had a considerable neutrophilic pleocytosis (41 and 59%). The remaining 2 cases had a mixed pleocytosis and a history compatible with the chronic form of the pathology, according to the history and in line with literature (1, 15). These two dogs were also the only ones where the CSF results might be affected by a pre-referral treatment, since a CSF analysis highly compatible with SRMA was found in all others pre-treated dogs. In our population, therefore, a pre-referral treatment did not constitute a diagnostic issue.

Breed distribution in our study reflects what has been previously described (1, 7, 8, 12). Multiple breeds were affected, however Boxers, mixed breed dogs, Bernese Mountain Dogs, Weimaraners and Beagles were overrepresented. Therefore, when clinically consistent, SRMA should be considered as a differential diagnosis regardless of breed. The median age (10 months), the range of age at first presentation (3–67 months), and the absence of a sex predisposition registered in our population reflect what has already been reported in the literature (1, 8). Twenty-five percent of dogs were not pyrexic, indicating that a normal temperature does not rule out SRMA as a differential diagnosis, as has previously been reported (16).

The first aim of our study was to investigate the MRI characteristics of the cervical spine in dogs diagnosed with SRMA. MRI showed abnormalities in 98.6% of cases, suggesting its considerable usefulness. Most of these cases had MRI features suggestive of meningeal inflammation (87.1%), with the cervical site most often affected being the tract between C2-C3 and C4. This finding could help during the acquisition of MRI of the cervical spine. The loss of signal intensity at the borders of the field of view in LF MRI could have biased this result, which therefore has to be considered cautiously. However, even in the studies conducted with HF MRI and characterized by a more homogeneous field of view, the aforementioned tract was the most typical area of contrast agent uptake.

Besides meningeal enhancement, spinal cord parenchymal enhancement was seen in 14.3% of our cases. Median duration of clinical signs before referral of these cases was 9.5 days (IQR 11.25; range 3–60 days) and most of them (8/10) showed deficits at neurological examination, consistent with the reported involvement of the motor and proprioceptive systems in the more chronic form of SRMA (1). However, no parenchymal contrast agent uptake was seen in the remaining 16 cases showing neurological deficits consistent with a cervical localization. Therefore, contrast agent uptake seems to be less useful in detecting parenchymal inflammation than meningeal inflammation, most likely for the less conspicuous vascularization of the parenchyma, which is provided by branches arising from the diffuse plexus present on the surface of the spinal cord (17). Moreover, we cannot exclude an overinterpretation of the neurological findings: sometimes large breed dogs with severe cervical pain show, due to the pain itself, an abnormal gait, that may be misinterpreted as ataxic or paretic.

The prevalence of enhancement of the synovium of the articular facets and of the paravertebral muscles in this study was the same for both conditions (48.6%). In veterinary literature SRMA and concomitant immune-mediated polyarthritis or immune-mediated myositis have been reported (11, 12). In one study, 46% of dogs with immune-mediated polyarthritis (IMPA) and spinal hyperesthesia had a CSF analysis consistent with SRMA (12). In that study, none of the dogs with concurrent SRMA and IMPA presented swelling of the appendicular joints or lameness, but inflammation was observed in synovial fluid analysis in 25–100% of the joints evaluated in each dog. As such, synovial fluid analysis has been suggested as a routine procedure in dogs with SRMA (12). The high incidence of enhancement of the synovium of the cervical articular facets detected on MRI in our study reinforces the supposition that polyarthritis could be more frequent than clinically expected.

It has been hypothesized that the pathogenesis of myositis in SRMA may include extension of the meningeal inflammation to the paraspinal muscles through spinal nerve roots (13). All but 4 cases with radicular enhancement in our study had muscular enhancement too, in agreement with this hypothesis. On the other hand, the majority of cases with muscular enhancement did not present a concomitant radicular contrast uptake and so the spread of inflammation through arteries could be hypothesized, which in addition would explain the presence of enhancement in the synovium of the articular facets, and hence the presumed cervical arthritis. In support of this hypothesis, arteritis of variable extent was found in several organs, including muscles, in a histopathologic study of SRMA (11).

In a previous report on inflammatory spinal cord diseases, muscular hyperintensity was seen in most of the cases and was detected better in STIR than in T2W images (13). The suppression of the signal of the adipose tissue ventral to the vertebrae in STIR sequences allows easier identification of muscular inflammation (13). We have seen a similar feature in our population, supporting the inclusion of STIR sequences in the MRI protocol in cases with suspected SRMA to highlight a potential muscular extension of the inflammation.

Contrast enhanced T1W images did not seem to provide more information than T2W images in the detection of idiopathic inflammatory myopathies in humans and dogs (18, 19). Our study identified a similar pattern; the administration of contrast agent always resulted in an enhancement in the T1W sequences in the areas of muscle showing T2W/STIR hyperintensity and never enabled the identification of further areas of putative muscular inflammation. However, other conditions, such as myonecrosis, fatty infiltration, rhabdomyolysis or soft tissue tumors with central necrosis, are associated with increased signal intensity on T2W images and can therefore resemble myositis (19, 20). Consequently, the acquisition of post-contrast T1W images remains essential in the MR investigations of presumed myositis.

Denervated muscles may present T1W contrast enhancement too (21). MRI can help in differentiating myositis from denervation as the former often results in multifocal changes while the latter causes diffuse changes affecting the entire muscle (19). We presume that our findings are consistent with myositis as the majority of cases had multifocal muscular enhancement and, when focal enhancement was found, it never affected the entire muscle.

The post-processing subtraction technique was a considerable aid in detecting subtle meningeal contrast enhancement. The utility of this technique at the spinal level is reported, especially for areas of contrast enhancement that are close to fat, whose bright signal tends to hide pathologic lesions (22, 23). Our findings fully support its application; we would recommend the use of subtraction on MRI of suspected SRMA cases, as the meninges are contiguous to the epidural fat.

The second aim of the present study was to compare the diagnostic capabilities of LF and HF MRI. We did not find any statistical difference between LF and HF MRI regarding the conspicuity of contrast enhancement in the meninges, synovium of the articular facet, muscles or spinal cord parenchyma. It would therefore appear that LF MRI can be used during the work up of cases of SRMA, because the greater spatial resolution of HF MRI is not required for these tissues. LF MRI underperformed in the detection of nerve root inflammation, since the percentage of cases with nerve root enhancement seen in LF MRI was significantly lower than those seen with HF MRI (*p* = 0.046). The conspicuity of contrast media uptake decreases with decreasing field strength (24). This could explain why LF MRI is less useful for detecting enhancement in small structures such as nerve roots.

The acquisition of T1W FAT SAT sequences is generally performed only with HF MRI, because the chemical shift between fat and water is too small in LF MRI to achieve a selective chemical saturation of fat without also producing water saturation (25). Acquisition of these sequences is possible after administration of a contrast agent. The hyperintense signal of the contrast agent is then not confused with the hyperintense signal of the adipose tissue. Thanks to the suppression of the epidural fat, the contrast-enhancing meninges are easily detected when an inflammatory process is present (26). Spectral fat saturation substantially improves the visualization of muscle inflammation as well (19). In this, our findings are consistent with previous studies and benefited from this technique.

In some of our cases, meningeal enhancement was only easily detectable on standard T1W images through the application of the subtraction technique. In the subtraction process the magnetic resonance signal is removed from the final image so that the densities recorded are dependent upon the vascularity of the tissue (27). Since adipose tissue has a low vascularity, fat signal is effectively suppressed with this technique (27), explaining the improved detection of meningeal enhancement.

For satisfactory subtraction studies, the post-contrast T1W sequence should reproduce exactly the cross-sections of tissue shown on the initial unenhanced sequence. A perfect subtraction study is unachievable when any minor movement between pre- and post-contrast scans takes place (27). Minimizing the deleterious effects that movements (respiratory and cardiac) of the anesthetized patient have on images is then an essential issue in the acquisition of MRI (23). To reduce the effects of motion artifact, some studies may require dedicated scanning hardware or software. These are generally present in HF scanners used in veterinary medicine as they are made for the human medical market, but may not be present in LF systems which are typically designed for human extremities, in which motion artifacts are hardly an issue (28). Moreover, the prominence of signal due to motion is reduced by increasing the signal to noise ratio (SNR) which is lower in LF compared to HF MRI (23). Nevertheless, these LF MRI features seem not to have affected the usefulness of subtraction in our study, as this was more frequently useful in LF than in HF MRI. On the contrary, it can be hypothesized that the lower utility of subtraction in HF MRI could be due to the higher spatial resolution (23) and to the higher conspicuity of contrast agent uptake in a higher field strength (24). Anyway, no significant difference was found between the utility of subtraction in LF and HF MRI (*p* = 0.059), therefore its application is always recommended, regardless of the field used.

Comparisons between LF and HF MRI must be interpreted cautiously due to the difference in the number of cases. An overall greater number of cases would have led to a more powerful statistical analysis.

A crossmatch between MRI findings and CSF results was applied in an attempt to establish a possible correlation between the severity of the inflammation detected through CSF analysis and the features of the contrast uptake on MRI.

Meningeal enhancement was not predictive of an abnormal CSF (*p* = 0.260). Since the number of cases with normal CSF was very low (*n* = 2), future studies with a greater number of cases could re-assess this finding.

When only the LF MRI were considered, CSF cell count significantly increased between the group with MRI meningeal score 1 and the group with MRI meningeal score 2; yet when only HF MRI was considered no significant increase was found. Thus, with a LF scanner the more the meninges are inflamed the more the enhancement is visible, while a HF scanner permits identification of the enhancement regardless of the degree of inflammation shown by the CSF analysis. Once again, a possible explanation for this trend could be the higher spatial resolution of HF MRI (23). Moreover, when pooling all the cases from the different types of MRI, CSF cell count significantly increased between all three groups. Then, stronger was the degree of contrast agent uptake in MRI and significantly higher was the cell count at the CSF analysis (Figure 4A). This finding highlights a possible correlation between the degree of meningeal inflammation seen on CSF and on MRI, probably because the increased vascular permeability due to the arteritis can explain both contrast agent leakage and cellular migration from the vascular compartment. Glucocorticoids could reduce the WBC count in CSF by reducing the degree of inflammatory response (15, 29) and a similar effect can be hypothesized for NSAIDs. Cases that received these drugs in our study were split almost proportionally among the three groups based on MRI meningeal score and the majority received a single dose. We therefore believe that it is very unlikely that the statistical results were affected by prior treatments.

In 9 (12.8%) cases there was no meningeal enhancement in spite of an abnormal CSF. The lack of contrast enhancement in inflammatory conditions is well-described in the literature (30–34). It has been supposed there may be a threshold of inflammation above which the disruption of the blood-meningeal barrier is enough to permit the visualization of the contrast enhancement when meningitis is present (35). It is possible that in our 9 cases this threshold was not reached. Indeed, 2 of our dogs received NSAID treatments before referral, 1 received glucocorticoid treatment, and 1 received both. These treatments could have lowered the degree of inflammation below the threshold and justify the lack of contrast enhancement.

Eight of the 9 cases that showed no meningeal enhancement in spite of an abnormal CSF also had concomitant enhancement of the synovium of the articular facets and/or muscular enhancement. The CSF cell count of these dogs was significantly lower than the CSF cell count of cases with synovium of the articular facets and/or muscular enhancement as well as concurrent meningeal enhancement (*p* = 0.040) (Figure 5A), despite a greater percentage of cases belonging to the latter group received glucocorticoid and/or NSAID treatments before referral. Due to these results, we may speculate the existence of cases of SRMA in which the aberrant immune-mediated response is directed more toward non-neurological structures. Different forms of SRMA or, at least, different stages with different tissular targets could therefore exist and future studies are needed to investigate this hypothesis.

According to the initial search of the database, 2 dogs had a diagnosis of SRMA in spite of a normal CSF analysis. Case number 1 was a 22-month-old, female Bernese Mountain Dog, with a previous diagnosis of SRMA 15 months earlier, a neurologic examination suggestive of cervical pain, and meningeal enhancement on MRI. Chronic/relapsing cases may have normal CSF (7, 10, 36), so we believe that this dog was likely affected by a second episode of SRMA, according to the MRI findings.

Case number 2 was a 4-month-old, male Boxer with a history of cervical pain in the absence of deficits at neurological examination, consistent with SRMA. MRI showed enhancement of the synovium of two pairs of articular facets. This dog could then be affected by a primary polyarthritis with cervical involvement (12). Since a concurrent inflammation of the synovium of the articular facets has been observed in 48.6% of the cases of SRMA in our study, and since CSF analysis can be normal in SRMA (2, 36), we hypothesized that this dog might be affected by a form of SRMA with a concurrent polyarthritis in a stage in which the aberrant immune-mediated response currently does not involve neurological structures.

This study has several limitations, mainly related to its retrospective nature.

The main limitation of the present study is the lack of a common gold standard diagnostic test, mostly due to the long duration of the study, encompassing a 13-year period. The diagnosis of SRMA was made on the basis of a combination of clinical and diagnostic information. The risk that a few cases with other diseases mimicking SRMA were included does exist.

MRI protocols varied through the years and post-contrast transverse images at one or more cervical levels were absent in many studies. This situation might have underestimated the real involvement of the meninges and extra-neural tissue, which could then be greater than we have reported. However, our results are based on the intensity of the contrast enhancement rather than on its extent along the cervical spine, therefore they should not have been altered by incompleteness of the imaging studies. Regarding the interpretation of the images, further limitations of the present study were the lack of a control group and that the observers were not blinded about neurological examination and the results of the clinical-pathological investigations.

A histopathological confirmation of the inflammation is lacking for both neural and extra-neural tissues and the diagnosis of polyarthritis and myositis is always presumed. However, in human beings it is reported that enhancement of the facets' joint rim after contrast agent administration will establish a diagnosis of synovitis (37). Moreover, although a histopathological diagnosis remains the gold standard for the diagnosis of human dermatomyositis, in recent decades the extensive use of MRI has restricted the number of biopsies carried out (38, 39).

## Conclusions

Meningeal enhancement is the most frequent diagnostic imaging finding in dogs affected by SRMA, often accompanied by enhancement of the synovium of the articular facets or muscular enhancement. When meningeal enhancement is absent, less marked CSF abnormalities are usually present. Low-field and HF MRI both have a good diagnostic overall capability, although HF MRI enables a more thorough investigation thanks to specific sequences, particularly T1W FAT-SAT, which can readily show meningeal enhancement.

In conclusion, this study highlights the usefulness of MRI as a complementary tool to CSF analysis in the diagnostic work up of SRMA. Furthermore, this study suggests MRI could have a diagnostic role in cases with a normal CSF but a clinical presentation suggestive of this condition. Further prospective studies are needed to complete the description of MRI patterns in SRMA and to more reliably compare LF and HF MRI.

## Data availability statement

The original contributions presented in the study are included in the article/supplementary material, further inquiries can be directed to the corresponding author/s.

## Ethics statement

Ethical review and approval was not required for the animal study because retrospective study. Written informed consent for participation was not obtained from the owners because retrospective study on routine clinical work-up.

## Author contributions

MB designed the study and supervised the study. AV and MB contributed the cases and finalized the manuscript. CR and AM selected and analyzed the cases and wrote the first draft. BC provided the statistical analysis. All authors provided input into the final version of the manuscript, contributed to the article, and approved the submitted version.

## Conflict of interest

The authors declare that the research was conducted in the absence of any commercial or financial relationships that could be construed as a potential conflict of interest.

## Publisher's note

All claims expressed in this article are solely those of the authors and do not necessarily represent those of their affiliated organizations, or those of the publisher, the editors and the reviewers. Any product that may be evaluated in this article, or claim that may be made by its manufacturer, is not guaranteed or endorsed by the publisher.

## Abbreviations

SRMA: Steroid-responsive meningitis-arteritis
MRI: magnetic resonance imaging
CSF: cerebrospinal fluid
LF: low field
HF: high field
T1W: T1 weighted
T2W: T2 weighted
FAT SAT: fat saturation
STIR: Short tau inversion recovery.
